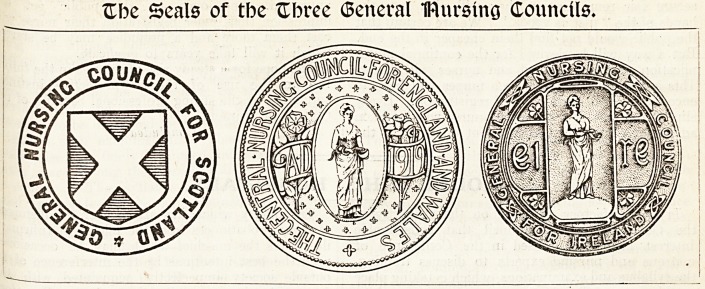# The Pay of the Nurse. VIII.—The Outlook for the Future
*Previous articles appeared March 5, 12, 26, and April 2, 9, 16 and 23.


**Published:** 1921-04-30

**Authors:** 


### The Seals of the Three General Nursing Councils.

### THE MATRONS' AND SISTERS' DEPARTMENT.

### THE PAY OF THE NURSE.*

### VIII.—The Outlook for the Future.

There is one strong weapon which nurses are entitled to use in their struggle for better pay. This is the refusal to enter any branch of their profession which falls below the rest in its prospects. It is perfectly legitimate for women trained in the exercise of their profession practically to boycott any class of employers who fail to realise their obligations. This can be done individually and it is a process which is perpetually going on.

In 1901 trained district nurses were being advertised for in London at 16s. rising to 18s. 6d. a week without board but with furnished lodgings and uniform; in the country at £40 a year with uniform, but without board or lodging. Always scarce at this figure district nurses gradually became unobtainable. They can now command from £160 to £175 a year. The same process has been going on in respect of probationers. Twenty years ago it was usual to advertise for them at a premium of £20. They can now command from £20 to £40 a year according to the class of institution, and the conditions they enjoy are immeasurably improved.

But, alas, in private work and in institutions the increase in pay which has taken place during the last twenty years is astonishingly less. Until lately nurses crowded into private practice disregardful of the fact that it no longer presents the same superiority in pay and conditions that it did at the beginning of the century over other branches of work. Where in 1901 trained nurses for institutions and nursing homes were offered from £30 to £40 a year with board, allowance for uniform and a small percentage on earnings, they are now being offered from £50 to £65. In other words, while the pay of district nurses and of probationers has more than trebled, that of private nurses sent out from institutes and nursing homes has not yet doubled. Fees have risen by one-third only. The moral is obvious, and there is reason to believe that the rush of the newly trained towards private work has considerably abated. It can be noted that a very small minority of the nurses trained in the foremost hospitals are now choosing private nursing as their career. The once coveted posts on the private staffs now often go a-begging.

We fear that there will always be a tendency towards a depreciation of pay in such forms of employment as happen to be popular, in comparison with other kinds of work which do not attract. That is so in all professions. But as a sense of corporate existence grows stronger nurses will not select their work haphazard but with intelligence, and increased pressure will be brought to bear on employers. It is to this natural and wholly beneficial form of combination that we must look to raise the rate of pay. Members of the College of Nursing have many opportunities of discussing the economic situation one with another, and nurses every day become better informed as to their prospects in different branches of work. Strength lies in this direction, and as time goes on the nurse who looks solely to her own hand will find her position untenable. Nurses as a body will come to appreciate the strong professional organisation of the College and its power to defend their interests. There was never a time when such defence was more needed than the present.

Danger lies in the great undisciplined and but partially trained tail to the profession. A thousand women are continually at hand willing to take lower pay, eager to pass themselves off as trained nurses, always full of arguments to convince doctors and patients alike that they are "just as good" as the real article. Unless the great body of trained nurses proceeds with patience and caution under the ægis of the College in their just determination to

* Previous articles appeared March 5, 12, 26, and April 2, 9, 16 and 23.

secure fair remuneration they will play into the hands of the "just as goods." We are certain that the public would not find them cheaper in the end. But a way will be devised for the continual multiplication of the untrained and nurses will suffer. This is the great danger which nurses will have to encounter in the early days of registration. Every slip on the part of a registered nurse will be taken advantage of to puff the excellent qualities of the untrained. And once let the public get the impression that under registration their nurse will cost them more and a prejudice may be started which it will take years to eradicate.

The hope for a steady rise of salaries in the future lies in the use of the self-protective instinct, supported by the strong professional backing of the College of Nursing.

(*Concluded.*)

## Figures and Tables

**Figure f1:**